# Jejunal Diverticulitis: A Rare Diverticular Disease of the Bowel

**DOI:** 10.7759/cureus.21386

**Published:** 2022-01-18

**Authors:** Venkata Vinod Kumar Matli, Viveksandeep Thoguluva Chandrasekar, Jason L Campbell, Chandana Karanam, Sathya Jaganmohan

**Affiliations:** 1 Internal Medicine, Christus Highland Medical Center, Shreveport, USA; 2 Gastroenterology, Augusta University Medical College of Georgia, Augusta, USA; 3 Interventional Radiology, Christus Highland Medical Center, Shreveport, USA; 4 Gastroenterology and Hepatology, Christus Highland Medical Center, Shreveport, USA

**Keywords:** diverticulitis mimic, complicated diverticulitis, duodenal diverticulosis, small bowel diverticulosis, jejunoileal diverticulitis

## Abstract

Diverticulosis is an out-pocketing of the bowel wall that can affect the small bowel through the large bowel. Small bowel diverticulosis is rare and not as common as colonic diverticulosis, which is an important diagnosis for hospitalizations. Moreover, jejunal diverticulosis is rare among cases of small bowel diverticulosis. Jejunal diverticulitis is one of the complications of jejunal diverticulosis that can be conservatively managed with antibiotics instead of surgery.

We report a case of a 41-year-old African American man who presented with vague epigastric pain and was diagnosed with adhesive jejunal diverticulitis upon contrast-enhanced computed tomography of the abdomen. The patient did not develop any life-threatening complications such as perforation or peritonitis, and recovered after conservative management with antibiotics. Adhesive jejunal diverticulitis with fat stranding was the distinctive finding in our patient, as he might have had multiple asymptomatic episodes.

Initial diagnostic modalities include radiography and contrast-enhanced computed tomography. Enteroclysis is the most reliable and accurate diagnostic modality, but is not available in all urgent settings. Recently, endoscopy has replaced radiological studies. Conservative management is adequate for uncomplicated cases of jejunal diverticulitis. However, surgical intervention is required in most cases of complicated jejunal diverticulosis, or mortality rates will be high.

## Introduction

Diverticulosis is a sac-like out-pocketing of the bowel wall that can affect the small bowel through the large bowel. Small bowel diverticulosis is rare and not as common as colonic diverticulosis, which is an important diagnosis for hospitalizations. Moreover, jejunal diverticulosis (JD) is rare among cases of small bowel diverticulosis and can occur anywhere in the small bowels. However, occurrence in the small bowel is rare and usually discovered through endoscopy and radiological imaging methods. The incidence of small bowel diverticulosis ranges from 0.3% to 1.3% in the general population and is 2.3% in autopsies [1], whereas the colonic diverticulosis prevalence rate ranges from 5% to 45% and is thus an important diagnosis for hospitalizations [2]. 

## Case presentation

**Figure 1:**
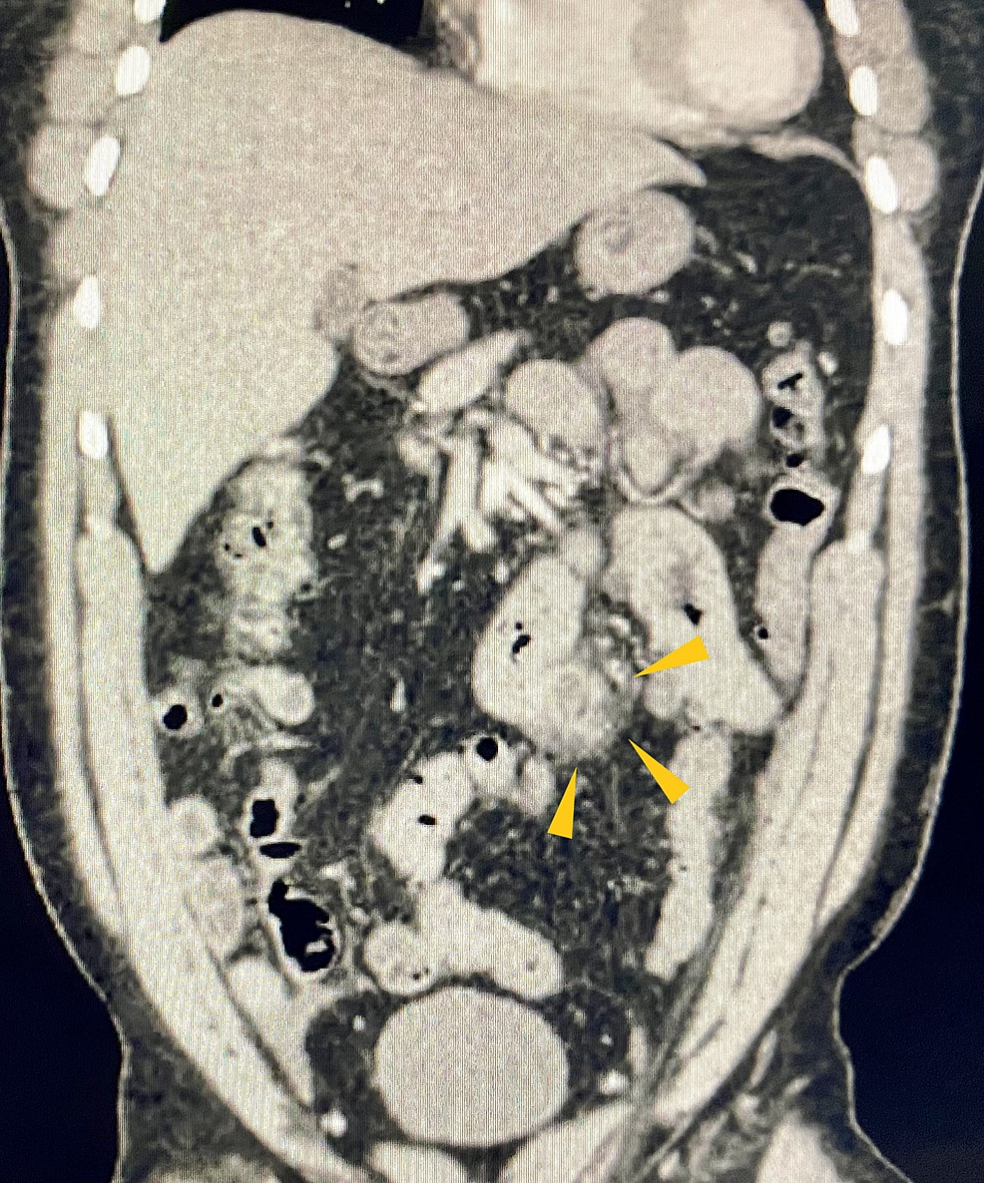
CT of the abdomen and pelvis with contrast (coronal view). Showing multiple diverticula (yellow triangle) involving the jejunum.

**Figure 2:**
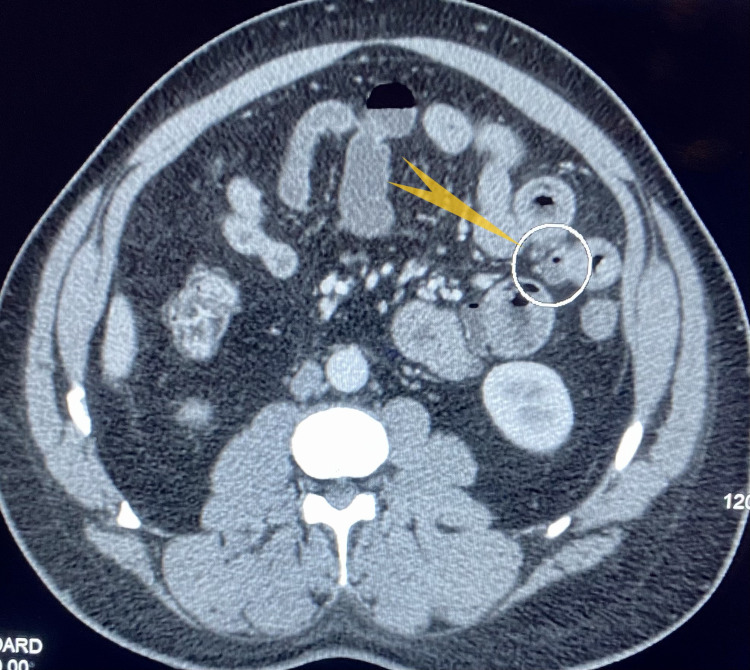
CT of the abdomen and pelvis with contrast. Showing jejunal diverticulum with thickened walls and marginal stranding of the mesenteric fat (pointed yellow arrow).

**Figure 3:**
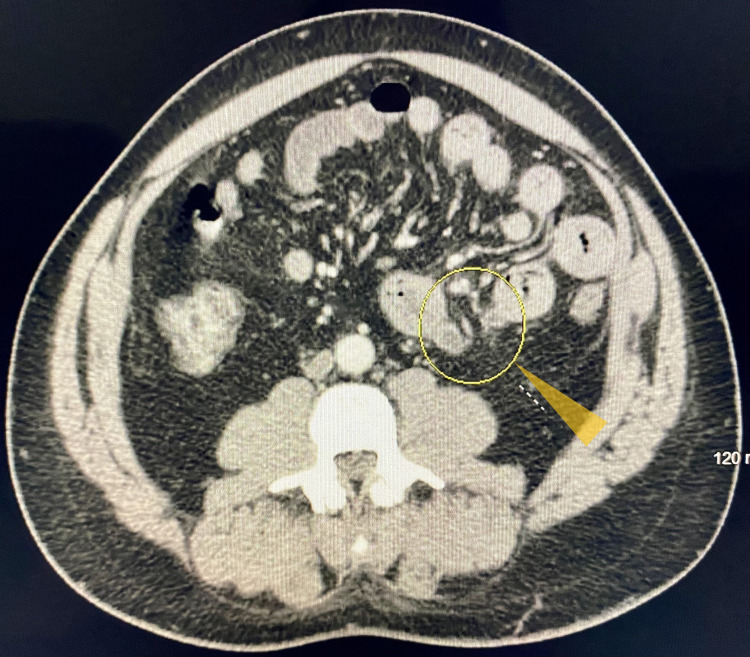
CT of the abdomen and pelvis with contrast. Showing an inflamed jejunal diverticulum in the central abdomen near the level of the aortic bifurcation and pelvic inlet (yellow triangle) consistent with jejunal diverticulitis.

We report a 41-year-old man with no significant medical history except essential hypertension. He presented with a sudden onset of epigastric and periumbilical abdominal pain with no associated symptoms such as nausea, vomiting, fever, chills, diarrhea, melena, or hematochezia. He had a history of intermittent, vague abdominal pain for which he did not seek medical help, as it has resolved on its own. The rest of his pertinent history included chronic tobacco use and occasional alcohol abuse. His vital signs were stable. Physical examination revealed tenderness in the upper abdomen but no rigidity or rebound tenderness. Bowel sounds were heard in all four quadrants. His labs including complete blood count and comprehensive metabolic panel, which included blood glucose levels, serum electrolytes and calcium, liver function tests, blood urea nitrogen and creatinine, were essentially within normal range. Plain abdominal radiography showed a non-obstructive bowel gas pattern, but was otherwise normal. Contrast-enhanced computed tomography (CT) of the abdomen and pelvis showed multiple diverticula involving the jejunum (Figure 1) with thickened walls, marginal stranding of the fat (Figure 2). A small amount of fluid was observed in the inferior margin of the right paracolic gutter and several small bowel loops. Findings were consistent with jejunal diverticulitis with adjacent fat stranding (Figure 3). The patient was administered intravenous piperacillin/tazobactam. General surgery was on board; however, the patient improved significantly with conservative management with antibiotics and did not require surgical intervention within 48 hours and was discharged. The gastroenterologist recommended outpatient follow-up and patient was clinically doing well during the follow-up clinic visit.

## Discussion

The first case of small bowel jejuno-ileal diverticulosis was published in 1794 [3]. JD is rare among small bowel diverticulosis cases, with duodenal diverticulosis (DD) being the more common type. However, patients with JD have a higher risk of developing complications than patients with DD [4,5]. In a retrospective study of 208 patients performed at three institutions over 23 years, 79% had duodenal diverticulosis, 18% had diverticulosis of the jejunum and ileum, and 3% had diverticulosis of the entire small bowel. Twenty percent of the patients developed complications such as perforations, hemorrhage, abscess formation, and bowel obstruction. The etiopathogenesis of the diverticula remained unclear. The probable hypothesis was that increased intraluminal pressure contributed to the diverticulum formation. Studies have reported that visceral neuropathy, which is observed in connective tissue disorders such as progressive systemic sclerosis and systemic lupus erythematosus, may cause increased intraluminal pressure, contributing to diverticulosis in these patients [6].

JD often presents with no symptoms, and the diagnosis is coincidental in 70% of patients. Thirty percent of patients present with nonspecific symptoms such as abdominal pain, malabsorption syndrome-related signs and symptoms that arise from complications such as diverticulitis, perforations with local or generalized peritonitis, obstruction, adhesions, abscess formation, gastrointestinal hemorrhage, and rarely, volvulus [7-9]. Thus far, there have been three interesting studies in which JD patients presented with abdominal pain that was found to have midgut volvulus secondary to a jejunal diverticulum. There were also reported cases with intestinal malrotation [7-9].

As the diagnosis of JD in most of the patients is incidental, they might suffer from diverticulitis long before they clinically present. Our patient might have had subclinical or asymptomatic episodes of diverticulitis before he was admitted to the hospital. Upon further interview, our patient also admitted that he had episodes of vague abdominal discomfort that resolved without any medical intervention, so he did not seek further medical help. 

Plain abdominal radiography is the initial diagnostic modality. However, the most accurate and reliable study is enteroclysis, which may not be possible to do in all urgent settings because of unavailability. Contrast-enhanced CT of the abdomen is the gold standard for providing useful diagnostic information [10]. In some patients, x-ray and CT of the abdomen may not be able to detect the diverticula that are located on the mesenteric side; in these cases, enteroclysis or endoscopy is required. Enteroscopy is another diagnostic option [11]. However, in recent years, endoscopy has replaced radiological methods and has been widely used for diagnosis [12]. A review of literature also showed that small bowel diverticulosis may be associated with hereditary neuromuscular disorders such as Cronkite-Canada syndrome, lipid storage disorders, Fabry’s disease, and sphingolipidosis [13].

Uncomplicated small bowel diverticulosis should be left untreated. Conservative management with intravenous antibiotics is adequate for diverticulitis, unless perforation or obstruction develops. Our patient responded to intravenous antibiotics and was discharged after two days of hospitalization. Complications usually occur in jejunal and ileal diverticular diseases and often require surgical interventions. Complications are rare in DD and usually improve with conservative management and surgical intervention may not be required [4].

## Conclusions

Unlike the more common colonic diverticulosis, JD is a rare form of bowel diverticulosis. Diagnosis of JD is usually incidental as the majority of patients are asymptomatic. Initial diagnostic modalities include radiography and contrast-enhanced computed tomography. Enteroclysis is the most reliable and accurate diagnostic modality, but is not available in all urgent settings. Recently, endoscopy has replaced radiological studies. Conservative management is usually adequate in uncomplicated patients. However, in complicated cases such as perforation, obstruction, abscess formation, and volvulus, emergent surgery is mandatory or the mortality rate will be high.

## References

[1] Kassahun WT, Fangmann J, Harms J, Bartels M, Hauss J (2007). Complicated small-bowel diverticulosis: a case report and review of the literature. World J Gastroenterol.

[2] Hughes LE (1969). Postmortem survey of diverticular disease of the colon. I. Diverticulosis and diverticulitis. Gut.

[3] De Peuter B, Box I, Vanheste R, Dymarkowski S (2009). Small-bowel diverticulosis:imaging findings and review of three cases. Gastroenterol Res Pract.

[4] Gurala D, Idiculla PS, Patibandla P, Philipose J, Krzyzak M, Mukherjee I (2019). Perforated Jejunal Diverticulitis. Case Rep Gastroenterol.

[5] Akhrass R, Yaffe MB, Fischer C, Ponsky J, Shuck JM (1997). Small-bowel diverticulosis: perceptions and reality. J Am Coll Surg.

[6] Krishnamurthy S, Kelly MM, Rohrmann CA, Schuffler MD (1983). Jejunal diverticulosis: a heterogenous disorder caused by a variety of abnormalities of smooth muscle or myenteric plexus. Gastroenterology.

[7] Jochmans I, Pirenne J (2016). Jejunal diverticulosis with midgut volvulus and intestinal malrotation. N Engl J Med.

[8] Hung WY, Huang CY (2020). Mesenteric volvulus from jejunal diverticulosis. N Engl J Med.

[9] Hu JL, Chen WZ (2012). Midgut volvulus due to jejunal diverticula: a case report. World J Gastroenterol.

[10] Coulier B, Maldague P, Bourgeois A, Broze B (2007). Diverticulitis of the small bowel: CT diagnosis. Abdom Imaging.

[11] Hortling N, Vahlensieck M, Schweikert HU (1995). [Clinical aspects and diagnosis of jejunal diverticulosis]. Aktuelle Radiol.

[12] Bach AG, Lübbert C, Behrmann C, Surov A (2011). [Small bowel diverticula - diagnosis and complications]. Dtsch Med Wochenschr.

[13] Chugay P, Choi J, Dong XD (2010). Jejunal diverticular disease complicated by enteroliths: report of two different presentations. World J Gastrointest Surg.

